# High Rates of Three Common *GJB2* Mutations c.516G>C, c.-23+1G>A, c.235delC in Deaf Patients from Southern Siberia Are Due to the Founder Effect

**DOI:** 10.3390/genes11070833

**Published:** 2020-07-21

**Authors:** Marina V. Zytsar, Marita S. Bady-Khoo, Valeriia Yu. Danilchenko, Ekaterina A. Maslova, Nikolay A. Barashkov, Igor V. Morozov, Alexander A. Bondar, Olga L. Posukh

**Affiliations:** 1 Federal Research Center Institute of Cytology and Genetics, Siberian Branch of the Russian Academy of Sciences, 630090 Novosibirsk, Russia; zytzar@bionet.nsc.ru (M.V.Z.); danilchenko_valeri@mail.ru (V.Y.D.); maslova@bionet.nsc.ru (E.A.M.); 2 Perinatal Center of the Republic of Tyva, 667000 Kyzyl, Russia; marita.badyhoo@mail.ru; 3 Novosibirsk State University, 630090 Novosibirsk, Russia; Mor@niboch.nsc.ru; 4 Yakut Scientific Centre of Complex Medical Problems, 677019 Yakutsk, Russia; barashkov2004@mail.ru; 5 M.K. Ammosov North-Eastern Federal University, 677027 Yakutsk, Russia; 6 Institute of Chemical Biology and Fundamental Medicine, Siberian Branch of the Russian Academy of Sciences, 630090 Novosibirsk, Russia; alex.bondar@mail.ru

**Keywords:** hearing loss, *GJB2*, founder effect, STR and SNP haplotypes, mutation age, Tuvinians, Altaians, Southern Siberia

## Abstract

The mutations in the *GJB2* gene (13q12.11, MIM 121011) encoding transmembrane protein connexin 26 (Cx26) account for a significant portion of hereditary hearing loss worldwide. Earlier we found a high prevalence of recessive *GJB2* mutations c.516G>C, c.-23+1G>A, c.235delC in indigenous Turkic-speaking Siberian peoples (Tuvinians and Altaians) from the Tyva Republic and Altai Republic (Southern Siberia, Russia) and proposed the founder effect as a cause for their high rates in these populations. To reconstruct the haplotypes associated with each of these mutations, the genotyping of polymorphic genetic markers both within and flanking the *GJB2* gene was performed in 28 unrelated individuals homozygous for c.516G>C (n = 18), c.-23+1G>A (n = 6), or c.235delC (n = 4) as well as in the ethnically matched controls (62 Tuvinians and 55 Altaians) without these mutations. The common haplotypes specific for mutations c.516G>C, c.-23+1G>A, or c.235delC were revealed implying a single origin of each of these mutations. The age of mutations estimated by the DMLE+ v2.3 software and the single marker method is discussed in relation to ethnic history of Tuvinians and Altaians. The data obtained in this study support a crucial role of the founder effect in the high prevalence of *GJB2* mutations c.516G>C, c.-23+1G>A, c.235delC in indigenous populations of Southern Siberia.

## 1. Introduction

Mutations in the *GJB2* gene (gap junction protein, beta-2, 13q12.11, MIM 121011) encoding transmembrane protein connexin 26 (Cx26) lead to nonsyndromic autosomal recessive deafness 1A (DFNB1A, MIM 220290) which is the most common form of hereditary hearing loss in many populations [1]. High prevalence of the *GJB2*-associated deafness makes the *GJB2* gene testing essential for the establishment of genetic diagnosis of hearing loss.

Over 400 deafness-associated variations in *GJB2* have been reported in the Human Gene Mutation Database (http://www.hgmd.cf.ac.uk) [2]. Specific ethno-geographic prevalence patterns were found for many of them [3,4,5]. For instance, variant c.35delG (p.Gly12Valfs*2) is prevalent in deaf patients of Caucasian origin [3,6]; c.235delC (p.Leu79Cysfs*3) is common in some Asian populations [4,7,8,9,10,11,12,13,14]; c.167delT (p.Leu56Argfs*26) is frequent in Ashkenazi Jews [15,16]; c.427C>T (p.Arg143Trp) is specific for population of Ghana (West Africa) and Peru (South America) [17,18]; c.71G>A (p.Trp24*) is widely spread in Indians and European Gypsies [19,20,21]; c.109G>A (p.Val37Ile) prevails in populations of Southeast Asia [5]; the splice donor variant c.-23+1G>A was found in many populations worldwide but extremely high prevalence of c.-23+1G>A was detected among Yakuts (Eastern Siberia, Russia) [22]; c.131G>A (p.Trp44*) was found with high frequency among descendants of ancestral Mayan population in Guatemala [23].

High prevalence of some major *GJB2* mutations in certain populations was explained by the founder effect as evidenced by conservation of haplotypes with closely linked markers. In some cases, analysis of genetic background of these mutations allowed to elucidate their approximate age and a presumable region of origin. The key role of the founder effect in prevalence of mutation c.35delG was established in numerous studies by analysis of the c.35delG-bearing haplotypes: this mutation first appeared approximately 10000–14000 years ago in the Middle East and/or the Mediterranean and then spread by human migrations throughout Europe and worldwide [24,25,26,27,28,29,30,31,32,33,34,35,36]. The conservation of haplotype bearing mutation c.167delT found in Ashkenazi Jews suggests a single origin of this mutation which began to spread since a presumed Ashkenazi population bottleneck [15,16]. Haplotype analysis of genetic markers flanking the *GJB2* gene showed that a high rate of mutation c.71G>A (p.Trp24*) common for Indians is most probably due to the founder effect, and the age of this mutation was calculated as 7880 years [20]. Contribution of the founder effect in extremely high rate of mutation c.-23+1G>A among Yakuts (Eastern Siberia, Russia) was evidenced by the c.-23+1G>A haplotype analysis, and the age of this mutation was estimated at approximately 800 years [22]. Common haplotype was established for specific mutation c.131G>A (p.Trp44*) found in individuals from Guatemala suggesting a single founder from ancestral Mayan population [23]. The founder effect was also suggested in high prevalence of mutation c.235delC in East Asians (China, Japan, Korea), Mongolians (Mongolia), and Altaians (Southern Siberia, Russia) but there were only a few studies of the c.235delC-bearing haplotypes to support this hypothesis [8,9,14,37,38,39]. Additionally, Yan et al. (2003) proposed that c.235delC has probably derived from a founder mutation approximately 11500 years ago in the Lake Baikal region and spread to some Asian regions through subsequent migrations [38]. A haplotype block specific to East Asians with the c.109G>A (p.Val37Ile) mutation was found among deaf patients of Chinese, Japanese, Vietnamese, and Philippines ancestry and the age of p.Val37Ile in this Asian cohort was estimated at approximately 300 generations [40]. Shinagawa et al. (2020) confirmed the founder effect in origin of six *GJB2* mutations frequently observed in Japanese hearing loss patients (c.235delC, p.Val37Ile, p.[Gly45Glu;Tyr136*], p.Arg143Trp, c.176_191del, and c.299_300delAT) and estimated the year at which each mutation occurred: c.235delC—around 6500 years ago, p.[Gly45Glu;Tyr136*]—around 6000 years ago, p.Arg143Trp—around 6500 years ago, c.176_191del—around 4000 years ago, c.299_300delAT—around 7700 years ago, and p.Val37Ile - around 14500 or 5000 years ago [39].

In our recent study, we evaluated the spectrum and frequency of the *GJB2* gene variants in a large cohort of deaf Tuvinian patients and the ethnically matched controls from the Tyva Republic (Southern Siberia, Russia) [41]. A striking finding was a high prevalence of rare specific variant c.516G>C (p.Trp172Cys) in the *GJB2* gene accounting for 62.9% of all mutant *GJB2* alleles found in Tuvinian patients and having carrier frequency of 3.8% in controls. Other frequent *GJB2* mutations found in Tuvinian patients were c.-23+1G>A (27.6%) and c.235delC (5.2%). The c.235delC was previously found as a major *GJB2* mutation in Altaians living in the Altai Republic (Southern Siberia, Russia) neighboring the Tyva Republic [10]. In our recent study on enlarged cohort of Altaian deaf patients, the proportion of c.235delC, c.516G>C, and c.-23+1G>A among all mutant *GJB2* alleles found in Altaian patients was estimated as 51.9%, 29.6%, and 14.8%, respectively [42].

High rate of three *GJB2* mutations c.516G>C, c.-23+1G>A, and c.235delC in Tuvinians and Altaians implies a crucial role of the founder effect in their prevalence in indigenous populations of Southern Siberia. In this study we test a presumable common origin of each of these *GJB2* mutations by analysis of haplotypes bearing c.516G>C, c.-23+1G>A, and c.235delC.

## 2. Materials and Methods

### 2.1. Subjects

The pathogenic contribution of the *GJB2* mutations to deafness and their carrier frequencies were evaluated in our preliminary studies in indigenous populations of Southern Siberia (Tuvinians and Altaians) and three *GJB2* mutations (c.516G>C, c.-23+1G>A, c.235delC) were found to be common [10,41,42]. For the analysis of haplotypes bearing these mutations, we recruited in total 28 unrelated deaf patients who were homozygous for c.516G>C (seventeen Tuvinians and one Altaian), for c.-23+1G>A (six Tuvinians) or for c.235delC (four Altaians). The ethnically matched control samples were represented by 117 unrelated healthy individuals without mutations c.516G>C, c.-23+1G>A, and c.235delC (62 Tuvinians and 55 Altaians).

The study was conducted in accordance with the Declaration of Helsinki, and the protocol was approved by the Bioethics Commission at the Institute of Cytology and Genetics SB RAS, Novosibirsk, Russia (Protocol No. 9, 24 April 2012).

### 2.2. STRs and SNPs Genotyping

**Figure 1:**
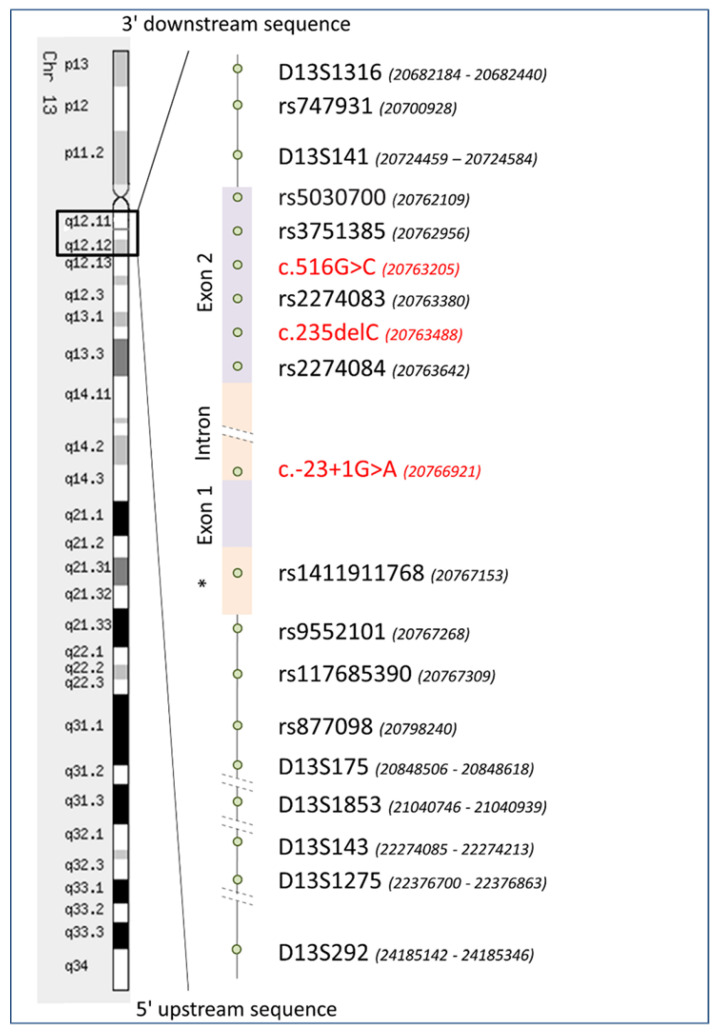
Schematic presentation of the *GJB2* gene structure and localization of genetic markers (seven STRs and nine SNPs) which were used for the reconstruction of haplotypes for *GJB2* mutations c.516G>C, c.-23+1G>A, and c.235delC. These mutations are marked by red color. *—basal (core) promoter (128 bp). Positions of genetic markers (shown in brackets) were defined according to GRCh37.p13 Genome Assembly (https://www.ncbi.nlm.nih.gov/assembly/GCA_000001405.14).

To determine common haplotypes for each of three major *GJB2* mutations c.516G>C, c.-23+1G>A, c.235delC, we performed genotyping of seven Short Tandem Repeats (D13S1316, D13S141, D13S175, D13S1853, D13S143, D13S1275, D13S292) flanking the *GJB2* gene and nine Single Nucleotide Polymorphisms (rs747931, rs5030700, rs3751385, rs2274083, rs2274084, rs1411911768, rs9552101, rs117685390, rs877098) intragenic and flanking the *GJB2* gene both in 28 unrelated deaf patients homozygous for c.516G>C, c.-23+1G>A, or c.235delC and in 117 unrelated healthy individuals (62 Tuvinians and 55 Altaians) who were negative for these mutations. The location of analyzed genetic markers on chromosome 13 is presented in Figure 1. Two additional SNPs (rs11147592, rs9509086) were genotyped in homozygous patients only. The total length of the region flanked by distal markers D13S1316 (centromeric) and D13S292 (telomeric) was approximately 3.5 Mb. All primers and genotyping methods are summarized in Supplementary Table S1. Fragment analysis and Sanger sequencing were performed in the SB RAS Genomics Core Facility (Institute of Chemical Biology and Fundamental Medicine SB RAS, Novosibirsk, Russia).

### 2.3. Reconstruction of STR and SNP Haplotypes 

The reconstruction of the founder haplotypes from STRs and SNPs genotyping data and analysis of their frequencies were performed using Expectation–Maximization (EM) algorithm of the Arlequin 3.5.2.2 software [43]. The boundaries of haplotypes for each of three *GJB2* mutations were determined by observed linkage disequilibrium between the marker alleles and each mutation according to equation δ = (Pd−Pn)/(1−Pn), where δ is the measure of linkage disequilibrium, Pd is the marker allele frequency among mutant chromosomes, Pn is the frequency of the same allele among normal chromosomes [44].

### 2.4. Estimation of Mutations Age

Estimation of a mutation age is based on the expected decay of linkage disequilibrium between the mutation and alleles of surrounding genetic markers due to recombination (“genetic clock” concept). We applied two approaches for estimating the age of mutations c.516G>C, c.-23+1G>A, and c.235delC. The first was the DMLE+ v2.3 software method (Disequilibrium Mapping using maximum-Likelihood Estimation, DMLE+: http://dmle.org/) [45] which is based on multiple linked marker loci and uses the Markov Chain Monte Carlo algorithm for Bayesian estimation of the mutation age. The second, used when appropriate, was the single marker method based on intra-allelic variation of a single marker [46]. For calculation the mutation age by the DMLE+ software, the demographic parameters (population size, population growth rate, and proportion of population sampled) are required in addition to the haplotype data and the map distances among marker loci and mutations. Since population growth rates for Tuvinian and Altaian populations could not be reliably estimated because of very limited knowledge of demographic variation of these populations along their history, we analyzed the haplotype data using several plausible growth rates: 0.05, 0.1, and 0.2. The parameter “proportion of population sampled” for each of three mutations (c.516G>C, c.-23+1G>A, c.235delC) was calculated on the basis of our previous data [10,41,42]. The contemporary population sizes for Tuvinians and Altaians according to the 2010 census were 249299 and 68814 peoples, respectively.

The estimation of the mutation age by the single marker method was performed using algorithm proposed by [46]:

g = log[1 − Q/(1 − Pn)]/log(1 − Ѳ)(1)

where g is the number of generations passed from the moment of the mutation appearance to the present; Q is the share of mutant chromosomes unlinked with the founder haplotype; Pn is the population frequency of allele included in the founder haplotype, and Ѳ is the recombinant fraction calculated from physical distance between marker and mutation (under the assumption that 1 cM = 1000 kb). To avoid possible underestimation of a mutation age as suggested by [47,48,49], we also applied the Luria-Delbrűck correction [50] which takes into account the demographic parameters:

g_c_ = g + g_0_ (2)

g_0_= −(1/d) ln(Ѳf_d_) (3)

where d is population growth rate, also assuming f_d_ = e^d^/(e^d^−1) and f_d_ ≈ 1/d at small d values [47].

The duration of one generation (g) was considered to be 25 years.

### 2.5. Statistical Analysis

Two-tailed Fisher’s exact test with significance level of *p* < 0.05 was applied to compare allele frequencies between patients and controls.

## 3. Results

We assumed that the high prevalence of *GJB2* mutations c.516G>C (p.Trp172Cys), c.-23+1G>A, c.235delC in Tuvinians and Altaians is a consequence of the founder effect. To test whether all carriers of each of these mutations share a common haplotype, we performed genotyping of polymorphic genetic markers both intragenic and flanking *GJB2* gene (nine SNPs and seven STRs) in 28 unrelated individuals homozygous for c.516G>C (n = 18), c.-23+1G>A (n = 6), or c.235delC (n = 4) as well as in ethnically matched controls (62 Tuvinians and 55 Altaians). The choice of analyzed genetic markers was based on their physical location, their variability in Asian populations, and the availability of previously published data for other populations. Results of the STRs and the SNPs genotyping are summarized in Supplementary Table S2.

### 3.1. STR Haplotypes

Data on genotyping of seven STR markers (D13S1316, D13S141, D13S175, D13S1853, D13S143, D13S1275, D13S292) flanking the *GJB2* gene and encompassing approximately 3.5 Mb (Figure 1) were used to reconstruct STR haplotypes both in deaf patients homozygous for each *GJB2* mutations (c.516G>C, c.-23+1G>A or c.235delC) and in the ethnically matched controls. The boundaries of the shared STR haplotypes were determined by observed linkage disequilibrium between STR alleles and each mutation.

Three different haplotypes formed by specific alleles of five STRs (D13S1316, D13S141, D13S175, D13S1853, D13S143) with a length of approximately 1.6 Mb were found to be associated with mutation c.516G>C (Table 1) in Tuvinian patients and 39 STR haplotypes were reconstructed in Tuvinian control sample (data not shown). The 269-124-105-204-125 haplotype was the most common (67.9%) among mutant chromosomes bearing c.516G>C, while the frequency of this haplotype in normal chromosomes (1.6%) was significantly lower (*p* < 10^−14^) (Table 1).

Significant linkage disequilibrium was found between mutation c.-23+1G>A and the specific alleles of six STRs (D13S141, D13S175, D13S1853, D13S143, D13S1275, D13S292) encompassing approximately 3.5 Mb long chromosome region. Three and sixty-eight STR haplotypes were reconstructed in Tuvinian patients homozygous for c.-23+1G>A (Table 1) and in Tuvinian controls (data not shown), respectively. Significant differences (*p* < 10^−8^) were observed between frequency of the 124-105-204-125-208-209 haplotype predominantly found among all mutant chromosomes with c.-23+1G>A (83.3%) and its frequency among normal chromosomes in Tuvinian controls (5.4%) (Table 1).

The only haplotype found in all mutant chromosomes with c.235delC (Altaian patients) was 267-124-105-204-125-210 (D13S1316-D13S141-D13S175-D13S1853-D13S143-D13S1275) flanked by markers D13S1316 and D13S1275 (~ 1.7 Mb), whereas this haplotype was not detected on normal chromosomes in Altaian control sample (*p* < 10^−11^) (Table 1).

### 3.2. SNP Haplotypes

To thoroughly analyze the structure of haplotypes associated with specific *GJB2* mutations, we have genotyped nine SNPs: four SNPs flanking *GJB2* gene (rs747931, rs9552101, rs117685390, rs877098) and five intragenic SNPs (rs5030700, rs3751385, rs2274083, rs2274084, rs1411911768) (Figure 1) in patients homozygous for c.516G>C, c.-23+1G>A, or c.235delC and in the ethnically matched controls. Significant linkage disequilibrium was observed between each of three *GJB2* mutations and certain alleles of all analyzed SNPs.

The only haplotype T-C-C-A-G-T-G-T-C (rs747931-rs5030700-rs3751385-rs2274083-rs2274084-rs1411911768-rs9552101-rs117685390-rs877098) was found on all (100%) mutant chromosomes with c.516G>C in Tuvinian patients in contrast with normal chromosomes in Tuvinian controls where 24 different SNP haplotypes were reconstructed (data not shown) and frequency of haplotype T-C-C-A-G-T-G-T-C was estimated to be 2.17% (*p* < 10^−26^) (Table 2).

Two SNP haplotypes were present in Tuvinian patients homozygous for c.-23+1G>A, while 26 different SNP haplotypes were reconstructed in the Tuvinian controls (data not shown). The C-C-C-A-G-C-G-T-C haplotype was predominant in Tuvinian patients (91.7%), while its frequency in the Tuvinian controls was 5.3% (*p* < 10^−10^) (Table 2).

Only one SNP haplotype T-C-C-A-G-C-G-T-T was found in Altaian patients homozygous for c.235delC, while 22 different SNP haplotypes were identified in Altaian controls (data not shown). This haplotype was the second by frequency in the Altaian control sample and differences found between its frequency in Altaian patients (100%) and controls (15.9%) were insignificant (Table 2).

Comparative analysis of SNP haplotypes associated with each of three *GJB2* mutations (c.516G>C, c.-23+1G>A, or c.235delC) revealed three SNPs (rs747931, rs1411911768, and rs877098), whose allelic compositions clearly define the specificity of each of these haplotypes. Two of these SNPs, rs747931 and rs877098, are located distantly from the *GJB2* gene, while rs1411911768 is located in basal (core) promoter region (128 bp) of the *GJB2* gene (Figure 1). Allele T of rs1411911768 included in the common haplotype associated with c.516G>C in Tuvinian patients was present in all corresponding mutant chromosomes, while it was absent in common haplotypes for mutations c.-23+1G>A and c.235delC (Table 2). Allele C of rs747931 was detected in both c.-23+1G>A-associated haplotypes found in Tuvinian patients but it was absent in haplotypes associated with c.516G>C or c.235delC (Table 2). Variant T of rs877098 was only found in c.235delC haplotype in Altaian patients and in more rare c.-23+1G>A haplotype in Tuvinian patients, and it was absent in c.516G>C haplotype (Table 2).

Thus, the unique allelic combination of three SNPs (rs747931- // -rs1411911768- // -rs877098) was found for each of the three most frequent SNP haplotypes bearing *GJB2* mutations: T-T-C for c.516G>C, C-C-C—for c.-23+1G>A, and T-C-T—for c.235delC.

### 3.3. Age of Mutations c.516G>C, c.-23+1G>A, and c.235delC 

The common haplotypes found for each of the mutations c.516G>C, c.-23+1G>A, or c.235delC prevailing in indigenous peoples of Southern Siberia imply that each of them had descended from a single ancestor. We estimated the numbers of generations (g) and years (in assumption that g = 25 years) passed from the common ancestral mutation event for each of these mutations assuming several population growth rates (0.05, 0.1, and 0.2) by the DMLE+ v2.3 program, which is sensitive to demographic parameters, and based on an analysis of multiple linked marker loci included in appropriate haplotype [45]. The single marker method for the estimation of the mutation age is based on the linkage disequilibrium and the recombination fraction observed for the alleles of surrounding genetic markers [46]. This approach implies analysis of alleles of the most distal markers which manifest significant linkage disequilibrium, while marker alleles with complete linkage disequilibrium (all disease chromosomes carried the same allele) are considered to be uninformative [51].

The DMLE+ program yielded the following estimations of the age of mutation c.516G>C: 91–180 generations (2275–4500 years) with d = 0.05, 57–106 generations (1425–2650 years) with d = 0.1 and 31–55 generations (775–1375 years) with d = 0.2 (Table 3). For c.516G>C age estimation by the single marker method we used allele (125) of the distal STR marker D13S143 found in high linkage disequilibrium with c.516G>C (Supplementary Table S2) that resulted in 27 generations passed from the origin of c.516G>C (675 years). After the Luria–Delbrűck correction allowing to avoid possible underestimation of a mutation age due to demographic parameters [47,48,49], the age of c.516G>C increased at all population growth rates (d = 0.05, 0.1 or 0.2): 51 generations (1275 years), 46 generations (1150 years), 40 generations (1000 years), respectively.

The DMLE+ estimations of the age of mutation c.-23+1G>A with different d (d = 0.05, 0.1, and 0.2) gave 73–164 generations (1825–4100 years), 42–91 generations (1050–2275 years), and 29–54 generations (725–1350 years), respectively (Table 3). When the age of c.-23+1G>A was estimated by the single marker method using allele (209) of the most distal marker D13S292 (more than 3.4 Mb from c.-23+1G>A), the age of c.-23+1G>A was drastically reduced to 4 generations (100 years) or 14–17 generations (350-425 years) after the Luria–Delbrűck correction at various d (0.05, 0.1 and 0.2).

We were not able to estimate the age of c.235delC using the single marker method because of the lack of recombination in all markers included in STR and SNP haplotypes observed for c.235delC. Nevertheless, by using the DMLE+ program, the variations of the age of c.235delC were reasonably consistent, being 45–126 generations (1125–3150 years), 34–79 generations (850–1975 years), and 22–46 generations (550–1150 years) with d = 0.05, 0.1, and 0.2, respectively (Table 3).

## 4. Discussion

We found three *GJB2* mutations, c.516G>C, c.-23+1G>A, and c.235delC to be predominant in deaf Tuvinian and Altaian patients [10,41,42]. Tuvinians and Altaians are the indigenous Turkic-speaking populations of two neighboring federal subjects of the Russian Federation, the Tyva Republic (Tuva) and the Altai Republic, respectively, which are located in Southern Siberia. The Tyva Republic is bordered by Mongolia in the south and the east, whereas the Republic of Altai is bordered by Mongolia in the southeast, China in the south, and Kazakhstan in the southwest.

### 4.1. Ethnic History of Tuvinians and Altaians 

Tuvinians (Tuvans) live mainly in the Tyva Republic in Russia (249299 people in total according to the 2010 census), though relatively small groups of Tuvinians also live in the northern part of Mongolia and in the Xinjiang Uygur Autonomous Region of China [52,53]. Tuvinians are one of the most ancient Turkic-speaking peoples inhabiting Central Asia and the Sayan-Altai region. The name "Tuva" probably originates from a Samoyedic tribe (referred to the VII century Chinese sources as “Dubo” or “Tupo”) that populated the upper Yenisei river region. The location of Tuva in the geographical center of the Asian continent had a significant impact on the formation of its population because of the relations with residents of neighboring regions. At different times, Tuva was at the periphery of a powerful state of Huns (II century BC–I century AD) or was incorporated in the Ancient Turkic Khaganate (VI–VIII centuries), in the Uyghur Khaganate (VIII–IX centuries), in the Yenisei Kyrgyz Khaganate (IX–XII centuries), and also in the Mongol Empire (XIII–XIV centuries), which played an outstanding role in the history of the nomadic civilization and the ethno-political development of Central Asia and the Sayan-Altai region. These historical events had a certain impact on the consolidation of ancestral Tuvinian tribes and, ultimately, on their formation into a single ethnic group. At the end of the XIII–XIV centuries, the ethnic composition of Tuva population already included those groups that took part in the formation of the Tuvinian people: descendants of different Turkic-, Mongolic-, Ket-, and Samoyedic-speaking tribes [54,55]. 

The Altaians, indigenous inhabitants of the Altai Republic (68814 people in total according to the 2010 census), belong to two main ethnic groups originated from several ancient Turkic-speaking tribes: Southern Altaians (Altai-kizhi, Teleut, and Telengit) and Northern Altaians (Chelkan, Kumandin, and Tubalar) [56]. Southern Altaian language belongs to the Kipchak branch of Turkic language family whereas the Northern Altai languages are greater influenced by Samoyedic, Yeniseian, and Ugric languages. In the past, the Altai region, as well as Tuva, was conquered or influenced by powerful Turkic Khaganates as well as the Mongol Empire [56].

Thus, archaeological, linguistic, anthropological, and historical evidences indicate similarities in the ethnogenesis of Turkic-speaking Tuvinian and Altaians.

### 4.2. Common Haplotypes for c.516G>C, c.-23+1G>A, and c.235delC 

**Figure 2:**
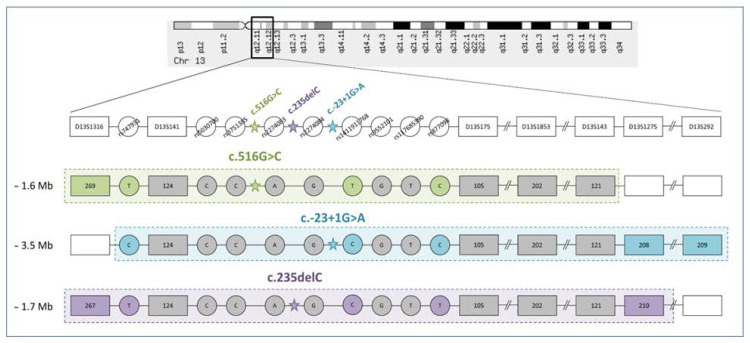
Schematic presentation of three common haplotypes bearing the *GJB2* mutations c.516G>C, c.-23+1G>A, or c.235delC. Locations of *GJB2* mutations and used genetic markers (seven STRs and nine SNPs) are shown at the top of the scheme. The STRs are indicated by rectangles, the SNPs - by circles. Founder haplotypes bearing mutations c.516G>C, c.-23+1G>A, c.235delC (spanning ~ 1.6 Mb, ~ 3.5 Mb, and ~ 1.7 Mb, respectively) are highlighted in dotted blocks. Identical alleles of genetic markers included in the haplotypes for each of *GJB2* mutations are shown in gray, while the alleles specific for corresponding mutations are indicated by different colors.

High rate of the *GJB2* mutations (c.516G>C, c.-23+1G>A, and c.235delC) in Tuvinians and Altaians implies a crucial role of the founder effect in their prevalence. Analysis of the genetic markers (seven STRs and nine SNPs intragenic and flanking the *GJB2* gene) surrounding mutations c.516G>C, c.-23+1G>A, and c.235delC revealed common haplotypes for each mutation, spanning ~ 1.6 Mb, ~ 3.5 Mb, and ~ 1.7 Mb, respectively (Figure 2). Moreover, we found the unique allelic combinations of three SNPs (rs747931- // -rs1411911768- // -rs877098) that were highly specific for each of the most frequent haplotypes bearing *GJB2* mutations (T-T-C for c.516G>C, C-C-C for c.-23+1G>A, and T-C-T for c.235delC). These combinations were absent or sufficiently less common in the control samples that allows to use them as additional markers for identification of major *GJB2* mutations in indigenous populations of Siberia.

### 4.3. The c.516G>C Mutation 

The *GJB2* variant c.516G>C (p.Trp172Cys, rs1302739538) accounts for 62.9% and 29.6% of all mutant *GJB2* alleles detected in deaf Tuvinian and Altaian patients, respectively, and the carrier frequencies of c.516G>C are 3.8% and 0.5% in the corresponding ethnically matched controls [41,42]. The c.516G>C substitution leads to a replacement of an aromatic non-polar tryptophan with a small polar cysteine at conservative amino acid position 172 (p.Trp172Cys) in the second extracellular loop of protein connexin 26 (Cx26). The c.516G>C meets the main criteria to be classified as pathogenic for autosomal recessive hearing loss based on the ACMG/AMP criteria [57] as specified by the Hearing Loss Expert Panel [58]. In our recent study [41], we suggested that this very rare *GJB2* mutation is endemic for Tuvinians living in the Republic of Tuva, since besides them c.516G>C was only found in Altaians from neighboring the Altai Republic (with less frequency) and in one deaf patient from Mongolia [59], and nowhere else in the world.

In this study, we obtained convincing evidence supporting the origin of mutation c.516G>C from a single ancestor. The common STR haplotype spanning about 1.6 Mb as well as the common internal SNP haplotype were identified in most of *GJB2* alleles carrying c.516G>C, and their frequencies in patients homozygous for c.516G>C were significantly different from controls. Interesting finding was a strong (100%) association of c.516G>C mutation with very rare allele T (A) of intragenic rs1411911768 (dbSNP: MAF A = 0.00002/3 TOPMED), which was found in Tuvinian and Altaian controls with sufficiently lower frequency (0.0565 and 0.0182, respectively) (Supplementary Table S2). We speculate that c.516G>C mutation could initially have arisen on the chromosome bearing rare allele of rs1411911768 in ancestors of these indigenous peoples (rather in Tuvinians, among whom c.516G>C is more prevalent) and reached current high prevalence as a result of the founder effect. The age of c.516G>C based on the single marker method was estimated to be 675 years or 1000–1275 years after the Luria–Delbrűck correction, whereas the dating of this event by the DMLE+ program led to wide time ranges (2275–4500, 1425–2650, or 775–1375 years ago) with different population growth rates (d = 0.05, 0.1, or 0.2, respectively). We tend to think that c.516G>C is rather a relatively “young” mutation since a fast population growth was probably intrinsic to Tuvinians in the past because of a traditionally large family size observed in contemporary Tuvinians. In addition, the prevalence of this mutation is very restricted. The most plausible scenario suggests that c.516G>C has arisen in the territory of Tuva as the result of a unique event after main formation of the Tuvinian ethnic group (which took place at the end of the XIII–XIV centuries) and then spread into the neighboring territory of Altai. Taking into account the complexity of ethnic history of Tuvinians, it remains unclear, who actually were the c.516G>C founders—different ancient Turkic- or Mongolic-speaking groups or other aboriginal peoples who lived there. The introduction of c.516G>C into Tuva territory with migration flows of ancient Mongolic-speaking groups is not consistent with the finding of c.516G>C in only one deaf patient from Mongolia [14,59] as well as with its absence in Mongolian patients living in China [60,61]. It is known that several nomadic Tuvinian groups roamed in the past across the territories of Tuva and Mongolia had remained in Mongolia when Tuva was separated from Mongolia to become under Russian protectorate after the breakup of the Qing Empire in 1911–1912 [54,55]. Since the ethnicity of examined deaf patients was not reported in the study by Tekin et al. [59], the question about the origin of c.516G>C in Mongolia remains open.

### 4.4. The c.-23+1G>A Mutation 

The proportion of the splice donor site mutation c.-23+1G>A reaches 27.6% of all mutant *GJB2* alleles in Tuvinian deaf patients [41] and 14.8% in Altaian patients [42]. Splice donor site *GJB2* variant c.-23+1G>A has been detected among deaf patients of different origin around the world [14,22,59,62,63,64,65,66,67]. The extremely high prevalence of c.-23+1G>A (up to 92.2% of all mutant *GJB2* alleles found in patients and carrier frequency reaching of 10.2%) observed in Yakuts, indigenous Turkic-speaking people living in the subarctic region of Russia (the Sakha Republic, Eastern Siberia), was explained by the founder effect in an isolated population and a probable selective advantage for the c.-23+1G>A heterozygotes in severe subarctic climate [22,67,68]. The c.-23+1G>A is also the most common mutation in deaf Mongolian patients from Mongolia [14,59].

To our knowledge, the haplotypes bearing c.-23+1G>A were analyzed only in a few studies [14,22,59,69]. Tekin et al. (2010) suggested diverse origins of c.-23+1G>A based on multiple c.-23+1G>A-associated haplotypes found in comparative analysis of seven Mongolian and three Anatolian Turkish c.-23+1G>A homozygous patients [59]. However, despite the fact that several different haplotypes were found to be associated with c.-23+1G>A in Mongolians, a single conserved haplotype (which appears to be a common haplotype in Mongolia) was identified in Turkish homozygous patients suggesting a single common ancestor with an intervening population bottleneck in the Turkish branch [59]. Barashkov et al. (2011) revealed the common origin of c.-23+1G>A in Yakuts (Eastern Siberia) by the reconstruction of 140 haplotypes bearing this mutation using eight polymorphic microsatellite markers flanking the *GJB2* gene and two intragenic SNP markers [22]. These findings are consistent with the founder effect hypothesis and support a common Central Asian origin of c.-23+1G>A since the Turkic-speaking ancestors of Yakuts migrated to the Eastern part of Siberia from their initial settlement in the Baikal Lake area under pressure of the Mongol expansion in XI - XIII centuries AD [70]. Solovyev et al. (2017) analyzed the c.-23+1G>A haplotypes in the sample of Yakut, Evenk, Russian, and Tuvinian deaf patients homozygous for c.-23+1G>A by using the same panel of SNPs (rs2313477, rs11841024, rs4769974, rs7994748, rs7987144, rs5030702, and rs1932429) as reported in the study by Tekin et al. [59] and revealed the reduced c.-23+1G>A haplotype diversity in the analyzed sample when compared with the haplotypes in Mongolians [69]. Interesting, that almost all examined patients (except one Yakut patient) in this study were homozygous for the allele T of intronic rs7994748 (*GJB2*) that is consistent with the studies by Grillo et al. (2015) and by Parzefall et al. (2017) in which the association of this rs7994748 allele with hearing loss was presumed [71,72]. In the study by Erdenechuluun et al. (2018) where five SNPs (rs747931, rs3751385, rs11147592, rs9509086, and rs9552102) were used for the c.-23+1G>A haplotype analysis in six Mongolian deaf patients, two c.-23+1G>A haplotypes were identified: major haplotype G-G-C-T-A (9/12 chromosomes) and a minor haplotype A-G-C-T-A (3/12 chromosomes) [14].

Our data on the common STR and SNP haplotypes for c.-23+1G>A found in Tuvinians evidence a single origin of this mutation and suggest the founder effect in its high prevalence in the Tyva Republic and neighboring territory of the Altai Republic. Based on the ethnic history of Tuvinians who experienced repeated influence of Mongolians at various stages of their ethnic formation [54,55], we speculate that c.-23+1G>A mutation can be introduced into Tuva by ancient Mongolic-speaking groups which were subsequently assimilated by the indigenous population of this region and then spread in Siberia by the migration flows. Our estimation of the c.-23+1G>A age yielded a wide range of 725–4100 years ago. This uncertainty could be probably attributed to a small size of the examined sample and an unclear population growth rate of Tuvinians in the past. Nevertheless, this estimation is consistent with previously reported age of c.-23+1G>A in the Sakha Republic (Yakutia) presumably introduced by Turkic-speaking ancestors of Yakuts approximately 800 years ago [22] further confirmed by the observed similarity of allelic composition of the common STR haplotypes in Tuvinian and Yakut patients homozygous for c.-23+1G>A (data not shown). Thus, our data support a proposed common Central Asian origin of mutation c.-23+1G>A and its further expansion defined by a specific population bottleneck at least throughout Siberia though further extensive studies in many populations are required to clarify this issue.

### 4.5. The c.235delC Mutation 

High prevalence of c.235delC mutation was found in our previous study in the Altai Republic [10] and was later confirmed in an extended cohort of Altaian deaf patients since alleles with c.235delC accounted for 51.9% of all mutant *GJB2* alleles found in patients and the carrier frequency of c.235delC reached 3.7% in Altaian population sample [42].

According to numerous studies, c.235delC mutation prevails in patients with hearing loss in Asian populations (China, Japan, Mongolia, Korea) [4,7,8,9,11,12,14,37,59,62]. The founder effect, implying the origin of c.235delC from a common ancestor, was suggested for the explanation of high prevalence of c.235delC in Asia. Several studies focusing on the analysis of the haplotypes bearing c.235delC confirmed this hypothesis despite the certain differences between the sets of used genetic markers [8,9,14,37,38,39]. Based on the STR and SNP analysis, we found only one haplotype associated with c.235delC in Altaian homozygous patients. It is worth noting that some SNPs included in c.235delC-associated haplotype in Altaians overlap with the SNP markers analyzed in other studies and alleles observed coincide with the ones found in Asian patients having c.235delC [9,14,37,38,39]. We suggest that these findings are in favor of a common c.235delC-associated haplotype at least among Altaians, Mongolians, Chinese, and Japanese and accordingly, in favor of the origin of c.235delC from one ancestor. Additional studies using a unified panel of markers are needed to clarify the question.

As for the age of c.235delC, as far as we know, this issue was elucidated in only two studies [38,39]. In the study by Yan et al. seven SNPs flanking this mutation were analyzed in deaf patients (in a total of 26 homozygotes and 19 heterozygotes for c.235delC) from various regions of Asia (China, Japan, Korea, Mongolia) and association of c.235delC with one core haplotype A-G-A-C (SNP2-V27I-E114G-SNP1), with a length of approximately 2.6 kb, was discovered [38]. The allele T of the most distant marker SNP6 (rs747931) located at ~ 63 kb from c.235delC was used to evaluate the mutation age resulting in 460 generations or approximately 11500 years (assuming 25 years per generation). Yan et al. speculated that c.235delC might have arisen in the Baikal area and then spread to Mongolia, China, Korea, and Japan through subsequent migration [38]. In recent study by Shinagawa et al. the c.235delC-associated haplotypes were analyzed in total of 20 Japanese patients homozygous for c.235delC [39]. Based on observed linkage disequilibrium for 5’SNP6 (rs4769920) located at ~ 265 kb from c.235delC, the occurrence of c.235delC mutation was estimated around 6500 years ago [39]. Notably, the single marker method was applied for c.235delC age estimation in both studies [38,39], while we could not estimate the age of c.235delC by this method due to the lack of recombination in c.235delC haplotype in Altaian patients. Our estimation by the DMLE+ led to the lower values of the age of c.235delC (22–126 generations or 550–3150 years at the different population growth rates). Although we do not exclude that c.235delC is really “younger” in Altaians in comparison with the data from these studies [38,39], these differences are more likely due to different methods of the age estimation, the panels of used genetic markers, the sample sizes, as well as uncertainty in growth rates of Altaian population along their history. Additionally, our data are based on the limited population of Southern Siberia (Altaians) whereas, for example, in the study by Yan et al. (2003) the samples from various countries (Mongolia, China, Japan, and Korea) were analyzed.

## 5. Conclusions

The common haplotypes specific for *GJB2* mutations c.516G>C, c.-23+1G>A, and c.235delC imply a single origin for each of them. A crucial role of the founder effect in high prevalence of these mutations in indigenous populations of Southern Siberia was established.

## Figures and Tables

**Table 1:** The frequencies of common STR haplotypes found among the chromosomes bearing c.516G>C, c.-23+1G>A, c.235delC in comparison with the normal chromosomes.

Haplotypes *	Frequency of Haplotypes		*x^2^*	*p*
	Mutant Chromosomes	Normal Chromosomes		
**Haplotypes for c.516G>C:** D13S1316-D13S141-D13S175-D13S1853-D13S143 (~ 1.6 Mb)				
**269-124-105-204-125**	0.6786	0.0161	79	<10^−14^
267-124-105-204-125	0.2857	0.2979	0.0093	0.5462
269-124-105-204-129	0.0357	0	0.67	0.1842
other haplotypes	0	0.6860	-	-
**Haplotypes for c.-23+1G>A:** D13S141-D13S175-D13S1853-D13S143-D13S1275-D13S292 (~ 3.5 Mb)				
**124-105-204-125-208-209**	0.8333	0.0538	53	<10^−8^
124-105-204-125-202-211	0.0833	0.0108	0.66	0.1695
124-105-204-125-210-209	0.0833	0.0472	0.011	0.4586
other haplotypes	0	0.8882	-	-
**Haplotypes for c.235delC:** D13S1316-D13S141-D13S175-D13S1853-D13S143-D13S1275 (~ 1.7 Mb)				
**267-124-105-204-125-210**	1.0	0	103	<10^−11^
other haplotypes	0	1.0	-	-

* The most common haplotypes are shown in bold.

**Table 2:** The frequencies of common SNP haplotypes found among the chromosomes bearing c.516G>C, c.-23+1G>A, c.235delC in comparison with the normal chromosomes.

Haplotypes *	Frequency of Haplotypes		*x^2^*	*p*
	Mutant Chromosomes	Normal Chromosomes		
**Haplotypes for c.516G>C:** rs747931-rs5030700-rs3751385-rs2274083-rs2274084-rs1411911768-rs9552101-rs117685390-rs877098				
**T-C-C-A-G-T-G-T-C**	1	0.0217	120	<10^−26^
other haplotypes	0	0.9783	-	-
**Haplotypes for c.-23+1G>A:** rs747931-rs5030700-rs3751385-rs2274083-rs2274084-rs1411911768-rs9552101-rs117685390-rs877098				
**C-C-C-A-G-C-G-T-C**	0.9167	0.0532	64	<10^−10^
C-C-C-A-G-C-G-T-T	0.0833	0.1540	0.047	0.4488
other haplotypes	0	0.7928	-	-
**Haplotypes for c.235delC:** rs747931-rs5030700-rs3751385-rs2274083-rs2274084-rs1411911768-rs9552101-rs117685390-rs877098				
**T-C-C-A-G-C-G-T-T**	1	0.1587	26	1
other haplotypes	0	0.8413	-	-

* The most frequent haplotypes are shown in bold. The SNP alleles specific for the common SNP haplotypes are highlighted by frames.

**Table 3:** Summarized results of the c.516G>C, c.-23+1G>A, c.235delC dating by the DMLE+ program.

Mutation	d	g (95% CI)	Age (95% CI)
c.516G>C	0.05 0.1 0.2	91–180 57–106 31–55	2275–4500 years 1425–2650 years 775–1375 years
c.-23+1G>A	0.05 0.1 0.2	73–164 42–91 29–54	1825–4100 years 1050–2275 years 725–1350 years
c.235delC	0.05 0.1 0.2	45–126 34–79 22–46	1125–3150 years 850–1975 years 550–1150 years

d—population growth rate; g—the number of generations; the age of mutation was calculated as g × 25 years.

## Supplementary Materials

The following are available online at https://www.mdpi.com/2073-4425/11/7/833/s1, Table S1: Primer sequences and methods for STRs and SNPs genotyping. Table S2: The allelic frequencies of STRs (D13S1316, D13S141, D13S175, D13S1853, D13S143, D13S1275, D13S292) and SNPs (rs747931, rs5030700, rs3751385, rs2274083, rs2274084, rs1411911768, rs9552101, rs117685390, rs877098) in deaf patients homozygous for mutations c.516G>C, c.-23+1G>A or c.235delC and in the control samples (Tuvinians and Altaians).
