# Supplementary material for: High Rates of Three Common GJB2 Mutations c.516G>C, c.-23+1G>A, c.235delC in Deaf Patients from Southern Siberia Are Due to the Founder Effect
Source: Genes (Basel). 2020 Jul 21;11(7):833. doi: 10.3390/genes11070833 (PMC7397271; doi:10.3390/genes11070833)
Supplement: Supplementary file 1 [file genes-11-00833-s001.zip › Supplementary files/Table S1.pdf]

Table S1. Primer sequences and methods for STRs and SNPs genotyping.

STRs	Primer sequences [a]	Methods of detection
D13S1316	F: 5’-CTACTGGGGAGGCTGG-3’ (NED)<br>R: 5’-CATGTCTCTGAATCGCTTTT-3’	Fragment analysis (500 LIZ)
D13S141	F: 5’-GTCCTCCCGGCCTAGTCTTA-3’<br>R: 5’-ACCACGGAGCAAAGAACAGA-3’ (6-FAM)	
D13S175	F: 5’-TATTGGATACTTGAATCTGCTG-3’<br>R: 5’-TGCATCACCTCACATAGGTTA-3’ (PET)	
D13S1853	F: 5’-TGTACATCTCTTCTTACATTCATGT-3’<br>R: 5’-CAGACTGGCACAAACTTAACTG-3’ (6-FAM)	
D13S143	F: 5’-CTCATGGGCAGTAACAACAAAA-3’<br>R: 5’-CTTATTTCTCTAGGGGCCAGCT-3’ (VIC)	
D13S1275	F: 5’-ATCACTTGAATAAGAAGCCATTTG-3’<br>R: 5’-CCAGCATGACCTTTACCAG-3’ (VIC)	
D13S292	F: 5’-TAATGGCGGACCATGC-3’ (PET)<br>R: 5’-TTTGACACTTTCCAAGTTGC-3’	
**SNPs**	**Primer sequences**	**Methods of detection**
rs2274083<br>rs2274084	835-F: 5’-TGCTTGCTTACCCAGACTCA-3’<br>835-R: 5’-CCTCATCCCTCTCATGCTGT-3’<br>or<br>CX26-F: 5’-TCTTTTCCAGAGCAAACCGC-3’<br>CX26-R: 5’-CTGGGCAATGCGTTAAACTGG-3’	Sanger sequencing
rs1411911768<br>rs9552101<br>rs117685390	Ex1-F: 5’-TCTTTTCCAGAGCAAACCGC-3’<br>Ex1-R: 5’-CTGGGCAATGCGTTAAACTGG-3’<br>or<br>Ex1-792-F: 5’-GCGTTCGTTCGGATTGGT-3’<br>Ex1-2239-R: 5’-CGGAAACAGACCCTCGTGAAGT-3’	Sanger sequencing
rs747931	F: 5’-TCGGCACCCCTACCTCCT-3’<br>R: 5’-TCAGACGACCAACCACCTAA-3’	PCR-RFLP analysis (*PstI*)
rs5030700<br>rs3751385	F: 5’-GGCTGGTGAAGTGCAACG-3’<br>R: 5’-GTAAGCAAACAAACTTTTGAAGTAG-3’	Sanger sequencing<br>PCR-RFLP analysis (*NheI*)
rs877098	F: 5’-ATGAGTATGGCAGATGATGTTATT-3’<br>R: 5’-CAAAAGTGGGCAAAGGTTTA-3’	PCR-RFLP analysis (*EcoRI*)
rs11147592	F 5’-GATTTCAGTGGCTTCCCTTT-3’<br>R 5’-CCAGGTCATACACCCTTCTACA-3’	Sanger sequencing
rs9509086	F 5’-GTCTTCCGCAGAATCCTATCAG-3’<br>R 5’-GGCTGTGGTCGTTGTCCTCT-3’	Sanger sequencing

[a] - Primer sequences for STRs genotyping were taken from the NCBI Probe Database (http://www.ncbi.nlm.nih.gov/probe) and the Ensembl Genome browser (http://www.ensembl.org). One from each primer pairs was marked with the different fluorescent dyes.
