# Supplementary material for: High Rates of Three Common GJB2 Mutations c.516G>C, c.-23+1G>A, c.235delC in Deaf Patients from Southern Siberia Are Due to the Founder Effect
Source: Genes (Basel). 2020 Jul 21;11(7):833. doi: 10.3390/genes11070833 (PMC7397271; doi:10.3390/genes11070833)
Supplement: Supplementary file 1 [file genes-11-00833-s001.zip › Supplementary files/Table S2.pdf]

Table S2. The allelic frequencies of STRs (D13S1316, D13S141, D13S175, D13S1853, D13S143, D13S1275, D13S292) and SNPs (rs747931, rs5030700, rs3751385, rs2274083, rs2274084, rs1411911768, rs9552101, rs117685390, rs877098) in deaf patients homozygous for mutations c.516G>C, c.-23+1G>A or c.235delC and in the control samples (Tuvinians and Altaians).

Tuvinians											Altaians				
**Genotype**		**c.516G>C / c.516G>C (n=14)**				**c.-23+1G>A / c.-23+1G>A (n=6)**				**Control (n=62)**	**c.235delC / c.235delC (n=4)**				**Control (n=55)**
**Markers**	**Allele**	**Number of alleles / frequency**	X^2	p	δ	**Number of alleles / frequency**	X^2	p	δ	**Number of alleles / frequency**	**Number of alleles / frequency**	X^2	p	δ	**Number of alleles / frequency**
D13S1316 [a]	259	0 / 0	-	-	0.0000	1 / 0.0833	2.1	0.0882	0.0833	0 / 0	0 / 0	-	-	0.0000	0 / 0
	263	0 / 0	0.42	0.2881	-0.0508	3 / 0.25	4.3	0.0329	0.2119	6 / 0.0484	0 / 0	0.086	0.6995	-0.0476	5 / 0.0455
	265	0 / 0	0.096	0.4389	-0.0333	0 / 0	0.069	0.6881	-0.0333	4 / 0.0323	0 / 0	-	-	0.0000	0 / 0
	267	8 / 0.2857	32.00	**$< 10^{-6}$**	-3.2177	8 / 0.6667	1	0.1550	-0.9683	103 / 0.8306	8 / 1	0.32	1.0000	**1.0000**	95 / 0.8636
	269	20 / 0.7143	71.00	**$< 10^{-7}$**	**0.7023**	0 / 0	0.0089	0.6255	-0.0420	5 / 0.0403	0 / 0	3	0.9322	-0.0092	1 / 0.0091
	271	0 / 0	0.42	0.2881	-0.0508	0 / 0	0.0019	0.5682	-0.0508	6 / 0.0484	0 / 0	0.0038	0.5601	-0.0784	8 / 0.0727
	273	0 / 0	-	-	0.0000	0 / 0	-	-	0.0000	0 / 0	0 / 0	3	0.9322	-0.0092	1 / 0.0091
	Total	28 / -				12 / -				124 / -	8 / -				110 / -
rs747931	C	0 / 0	11	**$< 10^{-4}$**	-0.5500	12 / 1	16	1	**1.0000**	44 / 0.3548	0 / 0	1.2	0.1338	-0.3012	25 / 0.2315
	T	26 / 1	11	1	**1.0000**	0 / 0	16	**$< 10^{-4}$**	-1.8182	80 / 0.6452	8 / 1	1.2	1	**1.0000**	83 / 0.7685
	Total	26 / -				12 / -				124 / -	8 / -				105 / -
D13S141 [a]	120	0 / 0	0.67	0.8158	-0.0081	0 / 0	2.1	0.9118	-0.0081	1 / 0.0081	0 / 0	-	-	0.0000	0 / 0
	124	28 / 1	2.30	**0.0100**	**1.0000**	12 / 1	0.54	1	**1.0000**	110 / 0.8871	8 / 1	0.88	0.1462	**1.0000**	109 / 0.9909
	126	0 / 0	1.80	0.0781	-0.1071	0 / 0	0.35	0.3140	-0.1071	12 / 0.0968	0 / 0	-	-	0.0000	0 / 0
	128	0 / 0	0.67	0.8158	-0.0081	0 / 0	2.1	0.9118	-0.0081	1 / 0.0081	0 / 0	0.88	0.1462	-0.0092	1 / 0.0091
	Total	28 / -				12 / -				124 / -	8 / -				110 / -
rs5030700	C	22 / 1	-	-	-	10 / 1	-	-	-	122 / 1	4 / 1	5.9	1.0010	**1.0000**	101 / 0.9902
	T	0 / 0	-	-	-	0 / 0	-	-	-	0 / 0	0 / 0	5.9	0.9623	-0.0099	1 / 0.0098
	Total	22 / -				12 / -				122 / -	8 / -				102 / -
rs3751385	C	26 / 1	7.2	1	**1.0000**	12 / 1	2.8	1	**1.0000**	90 / 0.7377	8 / 1	2.6	1	**1.0000**	72 / 0.6545
	T	0 / 0	7.2	**0.0009**	-0.3556	0 / 0	2.8	0.0321	-0.3556	32 / 0.2621	0 / 0	2.6	**0.0396**	-0.5278	38 / 0.3455
	Total	26 / -				12 / -				122 / -	8 / -				110 / -
rs2274083	A	28 / 1	1.1	1	**1.0000**	12 / 1	0.13	1	**1.0000**	115 / 0.9274	8 / 1	0.096	1	**1.0000**	99 / 0.9
	G	0 / 0	1.1	0.1513	-0.0783	0 / 0	0.13	0.4239	-0.0783	9 / 0.0726	0 / 0	0.096	0.4456	-0.1111	11 / 0.1
	Total	28 / -				12 / -				124 / -	8 / -				110 / -

Table S2 (continued)

Genotype		Tuvinians									Altaians				
		c.516G>C / c.516G>C (n=14)				c.-23+1G>A / c.-23+1G>A (n=6)				Control (n=62)	c.235delC / c.235delC (n=4)				Control (n=55)
**Markers**	**Allele**	**Number of alleles / frequency**	X^2	*p*	*δ*	**Number of alleles / frequency**	X^2	*p*	*δ*	**Number of alleles / frequency**	**Number of alleles / frequency**	X^2	*p*	*δ*	**Number of alleles / frequency**
rs2274084	A	0 / 0	6.3	**0.0017**	-0.2917	0 / 0	2.2	0.0550	-0.2917	28 / 0.2258	0 / 0	2	0.0658	-0.4286	33 / 0.3
	G	28 / 1	6.3	1	**1.0000**	12 / 1	2.2	1	**1.0000**	96 / 0.7742	8 / 1	2	1	**1.0000**	77 / 0.7
	Total	28 / -				12 / -				124 / -	8 / -				110 / -
rs1411911768	C	0 / 0	110	**$< 10^{-23}$**	-16.7143	12 / 1	0.026	1	**1.0000**	117 / 0.9435	8 / 1	1.1	1	**1.0000**	108 / 0.9818
	T	28 / 1	110	**$< 10^{-23}$**	**1.0000**	0 / 0	0.026	0.5158	-0.0598	7 / 0.0565	0 / 0	1.1	0.8685	-0.0185	2 / 0.0182
	Total	28 / -				12 / -				124 / -	8 / -				110 / -
rs9552101	G	28 / 1	0.65	1.022	**1.0000**	12 / 1	2.1	1.0040	**1.0000**	121 / 0.9918	8 / 1	-	-	-	110 / 1
	C	0 / 0	0.65	0.8133	-0.0083	0 / 0	2.1	0.9104	-0.0083	1 / 0.0082	0 / 0	-	-	-	0 / 0
	Total	28 / -				12 / -				122 / -	8 / -				110 / -
rs117685390	T	28 / 1	7.1	1	**1.0000**	6 / 1	0.8	1	**1.0000**	92 / 0.7541	8 / 1	2.9	1	**1.0000**	70 / 0.6364
	C	0 / 0	7.1	**0.0009**	-0.3261	0 / 0	0.8	0.1941	-0.3261	30 / 0.2459	0 / 0	2.9	**0.0321**	-0.5714	40 / 0.3636
	Total	28 / -				6 / -				122 / -	8 / -				110 / -
rs877098	C	26 / 1	8.8	1	**1.0000**	11 / 0.9167	1.6	0.0985	**0.7222**	84 / 0.6885	0 / 0	12	**0.0002**	-2.0556	74 / 0.6727
	T	0 / 0	8.8	**0.0003**	-0.4286	1 / 0.0833	1.6	0.0985	-0.3095	36 / 0.2951	8 / 1	12	1	**1.0000**	36 / 0.3273
	Total	26 / -				12 / -				122 / -	8 / -				110 / -
D13S175 [a]	101	0 / 0	0.83	0.1879	-0.0690	0 / 0	0.070	0.4678	-0.0690	8 / 0.0645	0 / 0	0.023	0.5193	-0.0891	9 / 0.0818
	103	0 / 0	13.00	**$< 10^{-4}$**	-0.5897	0 / 0	5.2	0.0054	-0.5897	46 / 0.3710	0 / 0	2.3	0.0539	-0.4667	35 / 0.3182
	105	28 / 1	26.00	1.0000	**1.0000**	12 / 1	11	1.0000	**1.0000**	55 / 0.4435	8 / 1	6.6	1.0000	**1.0000**	51 / 0.4636
	107	0 / 0	1.30	0.1217	-0.0877	0 / 0	0.20	0.3839	-0.0877	10 / 0.0806	0 / 0	0.0038	0.5601	-0.0784	8 / 0.0727
	109	0 / 0	0.67	0.8158	-0.0081	0 / 0	2.1	0.9118	-0.0081	1 / 0.0081	0 / 0	0.21	0.7523	-0.0377	4 / 0.0364
	111	0 / 0	0.01	0.5405	-0.0248	0 / 0	0.23	0.7573	-0.0248	3 / 0.0242	0 / 0	1.1	0.8685	-0.0185	2 / 0.0182
	113	0 / 0	0.67	0.8158	-0.0081	0 / 0	2.1	0.9118	-0.0081	1 / 0.0081	0 / 0	3	0.9322	-0.0092	1 / 0.0091
	Total	28 / -				12 / -				124 / -	8 / -				110 / -
D13S1853 [a]	196	0 / 0	-	-	0.0000	0 / 0	-	-	0.0000	0 / 0	0 / 0	3	0.9322	-0.0092	1 / 0.0091
	200	0 / 0	0.06	0.6645	-0.0164	0 / 0	0.66	0.8307	-0.0164	2 / 0.0161	0 / 0	3	0.9322	-0.0092	1 / 0.0091
	202	0 / 0	11.00	0.1513	-0.0783	0 / 0	0.13	0.4239	-0.0783	9 / 0.0726	0 / 0	0.70	0.2151	-0.2222	20 / 0.1818
	204	28 / 1	0.31	1.0000	1.0000	12 / 1	0.84	1.0000	**1.0000**	107 / 0.8629	8 / 1	1.6	1.0000	**1.0000**	81 / 0.7364
	206	0 / 0	0.24	0.3559	-0.0420	0 / 0	0.0089	0.6255	-0.0420	5 / 0.0403	0 / 0	0.0016	0.6036	-0.0680	7 / 0.0636
	208	0 / 0	0.67	0.8158	-0.0081	0 / 0	2.1	0.9118	-0.0081	1 / 0.0081	0 / 0	-	-	0.0000	0 / 0
	Total	28 / -				12 / -				124 / -	8 / -				110 / -

Table S2 (continued)

		Tuvinians									Altaians				
**Genotype**		**c.516G>C / c.516G>C (n=14)**				**c.-23+1G>A / c.-23+1G>A (n=6)**				**Control (n=62)**	**c.235delC / c.235delC (n=4)**				**Control (n=55)**
**Markers**	**Allele**	**Number of alleles / frequency**	X^2	***p***	***δ***	**Number of alleles / frequency**	X^2	***p***	***δ***	**Number of alleles / frequency**	**Number of alleles / frequency**	X^2	***p***	***δ***	**Number of alleles / frequency**
D13S143 [a]	123	0 / 0	0.67	0.8158	-0.0081	0 / 0	2.1	0.9118	-0.0081	1 / 0.0081	0 / 0	-	-	0.0000	0 / 0
	125	27 / 0.9643	0.61	0.2267	**0.6593**	12 / 1	0.44	1.0000	**1.0000**	111 / 0.8952	8 / 1	100	-	-	110 / 1
	127	0 / 0	0.09	0.4389	-0.0333	0 / 0	0.069	0.6881	-0.0333	4 / 0.0323	0 / 0	-	-	0.0000	0 / 0
	129	1 / 0.0357	0.01	0.5476	-0.0220	0 / 0	0.026	0.5158	-0.0598	7 / 0.0565	0 / 0	-	-	0.0000	0 / 0
	133	0 / 0	0.67	0.8158	-0.0081	0 / 0	2.1	0.9118	-0.0081	1 / 0.0081	0 / 0	-	-	0.0000	0 / 0
	Total	28 / -				12 / -				124 / -	8 / -				110 / -
D13S1275 [a]	198	0 / 0	-	-	0.0000	0 / 0	-	-	0.0000	0 / 0	0 / 0	3	0.9322	-0.0092	1 / 0.0091
	200	1 / 0.0357	0.24	0.6441	0.0036	0 / 0	0.069	0.6881	-0.0333	4 / 0.0323	0 / 0	-	-	0.0000	0 / 0
	202	4 / 0.1429	4.10	**0.0177**	-0.3454	1 / 0.0833	2.7	0.0431	-0.4388	45 / 0.3629	0 / 0	0.62	0.2340	-0.2088	19 / 0.1727
	204	0 / 0	0.67	0.8158	-0.0081	0 / 0	2.1	0.9118	-0.0081	1 / 0.0081	0 / 0	0.48	0.8086	-0.0280	3 / 0.0273
	206	4 / 0.1429	9.20	**0.0042**	0.1359	0 / 0	2.1	0.9118	-0.0081	1 / 0.0081	0 / 0	0.14	0.4123	-0.1224	12 / 0.1091
	208	16 / 0.5714	12.00	**0.0005**	0.4029	10 / 0.8333	13	**0.0028**	**0.7678**	35 / 0.2823	0 / 0	8	**0.0014**	-1.3913	64 / 0.5818
	210	0 / 0	5.70	**0.0028**	-0.2653	1 / 0.0833	0.45	0.2661	-0.1599	26 / 0.2097	8 / 1	51	**$<10^{-7}$**	**1.0000**	7 / 0.0636
	212	3 / 0.1071	0.15	0.3284	0.0456	0 / 0	0.070	0.4678	-0.0690	8 / 0.0645	0 / 0	1.1	0.8685	-0.0185	2 / 0.0182
	214	0 / 0	0.096	0.4389	-0.0333	0 / 0	0.069	0.6881	-0.0333	4 / 0.0323	0 / 0	1.1	0.8685	-0.0185	2 / 0.0182
	Total	28 / -				12 / -				124 / -	8 / -				110 / -
D13S292 [a]	205	16 / 0.5714	23.00	**$< 10^{-6}$**	**0.4790**	0 / 0	1.4	0.1089	-0.2157	22 / 0.1774	0 / 0	0.31	0.3288	-0.1579	12 / 0.1364
	207	0 / 0	8.00	**$< 10^{-5}$**	-0.3626	0 / 0	2.9	0.0302	-0.3626	33 / 0.2661	0 / 0	1.4	0.1137	-0.3333	22 / 0.25
	209	6 / 0.2143	0.17	0.3478	-0.0825	11 / 0.9167	18	**$< 10^{-4}$**	**0.8852**	34 / 0.2742	3 / 0.375	0.25	0.2912	0.1912	20 / 0.2273
	211	6 / 0.2143	0.25	0.3166	-0.0947	1 / 0.0833	1.3	0.1212	-0.2772	35 / 0.2823	5 / 0.625	0.88	0.1734	0.3889	34 / 0.3864
	Total	28 / -				12 / -				124 / -	8 / -				88 / -

[a] - Designation of the STR allele corresponds to its size in nucleotides; the maximum indices of linkage disequilibrium (δ) and statistically significant ($p < 0.05$) differences in allele frequencies between the c.516G>C, c.-23+1G>A, c.235delC homozygotes and the control samples are in bold.
